# Effect of Non-Surgical Periodontal Therapy on Systemic Inflammatory Markers, Glycemic Status and Levels of Proteinuria in Type 2 Diabetic and Non-Diabetic Patients With Chronic Periodontitis

**DOI:** 10.7759/cureus.44757

**Published:** 2023-09-06

**Authors:** S Gopalakrishnan Sundaram, T Ramakrishnan, Sneha G Krishnan, Keerthi V Narayan, Siva Shankar, G Kanimozhi

**Affiliations:** 1 Periodontology, Thai Moogambigai Dental College and Hospital, Dr MGR Educational and Research Institute, Chennai, IND; 2 Periodontics, Dental Clinic, Chennai, IND; 3 Dentistry, Sri Ramaswamy Memorial (SRM) Dental College and Hospital, Chennai, IND; 4 Oral and Maxillofacial Pathology, Axon Anaesthesia Associates, Hyderabad, IND; 5 Periodontology, Tamil Nadu Government Dental College and Hospital, Chennai, IND; 6 Dentistry, Private Dental Clinic, Chennai, IND

**Keywords:** functional urology, glycated hemoglobin (hba1c), periodontal risk factors, periodontal health status, glycaemic parameters, fasting blood glucose (fbg), massive proteinuria, diabetes type 2

## Abstract

Aims and objectives:* *The present study aimed to evaluate the effect of non-surgical periodontal therapy (NSPT) on systemic inflammatory markers, glycemic status, and levels of proteinuria in Type 2 diabetic and non-diabetic individuals with chronic periodontitis.

Methodology: A total of 120 patients, categorized into three groups of 40 each, were included in this randomized observational study. Group 1 comprised patients with chronic periodontitis; Group 2 had chronic periodontitis with controlled diabetes; and Group 3 represented patients with chronic periodontitis with uncontrolled diabetes based on fasting blood sugar (FBS) and glycated hemoglobin (HbA1c) levels. Periodontal clinical parameters like plaque index, gingival index, bleeding on probing, pocket depth, and clinical attachment levels were evaluated. Blood samples and urine samples were collected and assessed for the levels of FBS, HbA1c, total protein, albumin, globulin, and proteinuria. All parameters recorded at baseline and three months after non-surgical periodontal therapy were analyzed for statistical significance at p <.05 using SPSS Inc. Released 2007. SPSS for Windows, Version 16.0. Chicago, SPSS Inc.

Results: A significant reduction in the periodontal clinical parameters within the groups, except for the clinical attachment level in Group 1 patients (p = 0.05), was observed. Glycemic status revealed a significant reduction after non-surgical periodontal therapy (p < 0.001), and on intragroup comparison, the total protein, albumin, globulin, and microprotein blood and urine levels showed significance among the evaluated groups (p < 0.001).

Conclusion: Non-surgical periodontal treatment can effectively improve the periodontal and circulating inflammatory status. Results of our study showed improved glycemic control and a reduction in systemic inflammatory markers and proteinuria after performing non-surgical periodontal treatment in patients with type 2 diabetes.

## Introduction

Diabetes mellitus and periodontal diseases belong to the class of chronic inflammatory disorders with potential influence on the overall general health and well-being of billions of individuals worldwide. Evidence-based studies consistently reveal diabetes as a strong risk factor for increased severity of gingival and periodontal diseases or conditions [1,2]. Conversely, periodontitis worsens glycemic control in patients with diabetes and may induce severe complications in long-standing cases [3,4]. Many studies have been published describing the bi-directional interrelationship exhibited by diabetes and periodontal disease [5]. The shared susceptibility between diabetes and periodontitis shows that both conditions are linked through mechanisms such as an increase in inflammatory cytokines, adipokine, advanced glycated end products, and abnormal neutrophils. Elevations in the serum inflammatory and thrombotic mediators may significantly contribute to maintaining the levels of glycemic index in patients with periodontitis, specifically through the insulin resistance mechanism [6].

Elevated acute-phase inflammatory response markers, predominantly cytokines, were observed in the serum concentrations of the patients with type 2 diabetes, indicating that the presence of circulating inflammatory cytokines considerably modifies the risk for diabetes, cardiovascular disease, atherosclerosis, and premature birth of children with reduced body weight [7]. Consequently, it is very important to study the association between periodontal disease and total protein, serum albumin, and globulin levels, which reflect the general health status of an individual who may be at higher risk of developing inflammatory conditions or disorders. Another significant complication of diabetes is proteinuria, which is the excretion of protein in urine. It is evident that long-standing diabetes affects kidneys and filtration rates, which eventually lead to proteinuria [7].

Cytokines promote inflammation by increasing leukocyte infiltration in the juxta glomeruli apparatus and convoluted tubules, along with a substantial rise in the infiltration of chronic inflammatory leukocytes such as macrophages. Interleukin-6, a pleiotropic cytokine that is predominantly elevated in chronic inflammatory disorders, largely affects the extracellular matrix, increases fibronectin expression, and enhances endothelial permeability [8]. These findings show that there is an elevated systemic marker of inflammation in periodontitis patients. Most of the research has focused only on proving the bidirectional relationship between diabetes and periodontitis, while the complications of diabetes are left unexplored. Despite studies that have shown improved clinical symptoms and decreased levels of pro-inflammatory cytokines, chemokines, and glycemic levels following periodontal therapy, these observations lack extensive evidence to confirm the role of systemic inflammatory markers present in periodontitis and their influence on systemic diseases [9]. Thus, the present study aimed to evaluate the effect of non-surgical periodontal therapy on systemic inflammatory markers, glycemic status, and levels of proteinuria in Type 2 diabetic and non-diabetic patients with chronic periodontitis.

## Materials and methods

The study participants were carefully chosen from the periodontology outpatient department at Thai Moogambigai Dental College and Hospital, Chennai, India, based on the inclusion and exclusion criteria as follows: The study included patients diagnosed with chronic periodontitis with or without Type 2 diabetes mellitus who were clinically present with at least four teeth with probing depth (PD) ≥ 5mm at more than one site, a clinical attachment level (CAL) ≥ 5 mm, and bleeding on probing (BOP). However, patients with a recent history of periodontal surgeries, periodontal procedures in the past six months, patients on antibiotic therapy, pregnant patients, a history or habit of smoking or alcohol consumption, and those with less than 20 remaining natural teeth were excluded.

A total of 120 participants were selected and divided into three groups of 40 patients each. Group 1 comprised patients with chronic periodontitis; Group 2 comprised patients with chronic periodontitis with controlled type 2 diabetes mellitus; and Group 3 represented patients with chronic periodontitis with uncontrolled type 2 diabetes mellitus. The purpose of the study was explained to all the participants, and written informed consent was also obtained to ensure voluntary participation. All participants completed the study without any interference. Ethical committee approval for the study protocol was obtained from the University Ethics Committee (Dr. MGRDU/TMDCH/2013-14/12001), and the study was conducted under the Helenski Declaration of 1975, as revised in 2013.

Periodontal treatment and clinical measurements

All patients were subjected to a complete periodontal examination of all the surfaces on the tooth, excluding the third molar. Periodontal parameters like Plaque Index (Silness and Loe 1964), Gingival Index (Loe and Silness 1963), bleeding on probing (BOP), probing pocket depth, and clinical attachment level were evaluated using the UNC 15 probe.

Blood and urine samples were obtained after an overnight fast of 8 to 10 hours from all individuals at the time of assessment and three months after treatment. The patients received four sessions of non-surgical periodontal treatment using ultrasonic scales and Gracey's curettes within one month, comprising oral hygiene instructions, scaling, and root planing under local anesthesia. A professional plaque control program demonstrating methods of removal of supra-gingival plaque, the need for oral hygiene, and oral hygiene practices was accomplished twice a month over three months following periodontal therapy. During this observational period, participants were frequently questioned about changes in lifestyle, exercise, and diet, regular intake of diabetic medications, and the use of any anti-inflammatory or antibiotics for other illnesses. 

Sample preparation

**Figure 1:**
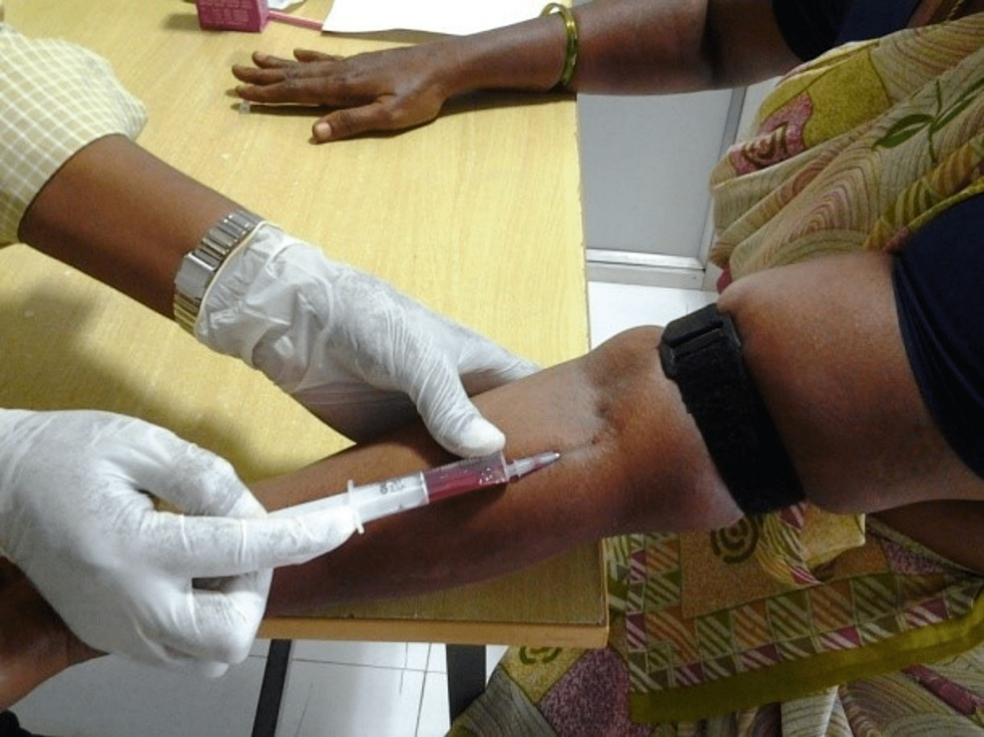
Image showing the collection of a venous blood sample from the study participant

**Figure 2:**
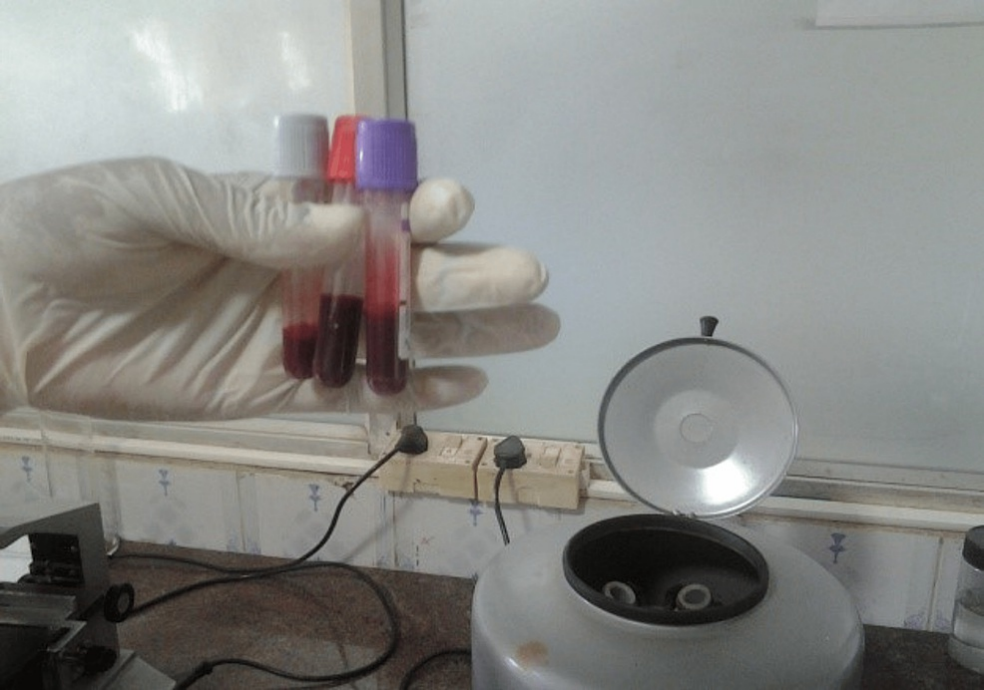
Image showing the preparation of sample tubes for centrifugation

**Figure 3:**
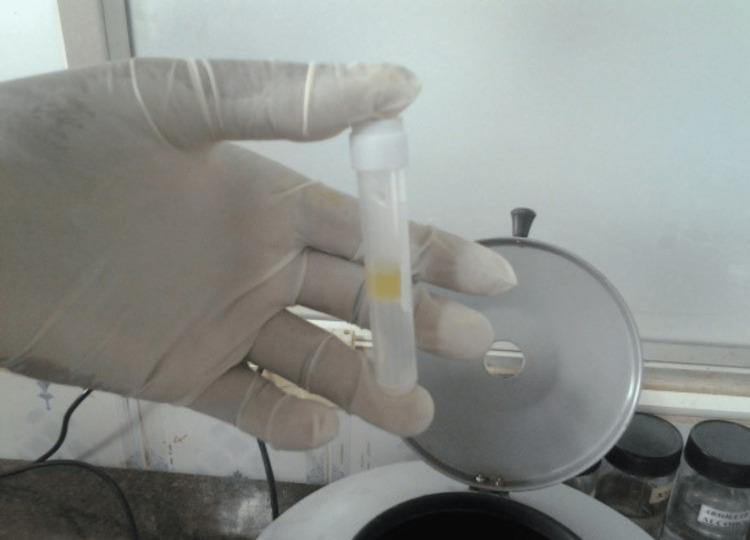
Image showing the supernatant obtained following centrifugation

Venous blood was collected from the patients in all three groups after an eight-hour overnight fast (Figure 1). The collected blood samples were separated, distributed in the respective test tubes, and then prepared for centrifugation (Figure 2). Following centrifugation, the supernatant was separated, collected in a separate tube to mix with the reagents (Figure 3), and measured using an atomic spectrophotometer. Fasting plasma glucose (blood sugar FBS) level was measured by the GOD-POD method, glycosylated hemoglobin (HbA1c) concentration by the Column method, total protein by the Biurette method, albumin by the Bromocresol green method, and micro-protein by the Pyrogollol method.

Statistical analysis

Statistical analyses were performed using SPSS Inc. Released 2007. SPSS for Windows, Version 16.0. Chicago, SPSS Inc. One-way analysis of variance (ANOVA) and paired T-tests were carried out to compare the study variables and analyze the statistical significance within and between the groups.

## Results

In the present study, statistically significant differences were observed within the groups (intragroup) in all the clinical parameters except for the clinical attachment level in Group 1 (p =0.05) (Table 1). On the intergroup comparison of clinical parameters, statistical significance was observed for the Plaque Index parameter only between Group 2 and Group 3 patients (p < .05) (Table 2).

**Table 1:** Table showing the intra-group comparison of the mean ± SD of clinical parameters at baseline and three months after treatment using a paired t-test *p<.05: Significant level

Groups	Plaque index Mean ± SD	Gingival index Mean ± SD	Bleeding on probing	Pocket depth Mean ± SD	Clinical attachment level Mean ± SD
Group 1					
Mean value at baseline	2.13 ± 0.69	2.43 ± 0.62	2.16 ± 0.68	3.84 ± 1.33	4.13 ± 0.79
Mean value after 3 months	0.80 ± 0.24	0.69 ± 0.26	0.92 ± 0.38	1.87 ± 1.06	2.97 ± 0.99
p-value	.003*	.0016*	.0089*	.0031*	.05
Group 2					
Mean value at baseline	2.11 ± 0.44	2.66 ± 0.37	2.16 ± 0.53	4.04 ± 1.02	4.36 ± 0.67
Mean value after 3 months	0.94 ± 0.08	0.67 ± 0.21	1.02 ± 0.31	1.88 ± 0.54	3.06 ± 1.05
p-value	.0039*	.0001*	.0075*	.00121*	.0018*
Group 3					
Mean value at baseline	1.66 ± 0.55	2.46 ± 0.77	2.11 ± 0.59	4.80 ± 0.57	4.03 ± 0.47
Mean value after 3 months	0.60 ± 0.20	0.94 ± 0.34	0.79 ± 0.29	1.99 ± 0.71	2-73 ± 1.12
p-value	.0052*	.0046*	.0039*	.0003*	.00363*

**Table 2:** Table showing the inter-group comparison of the mean ± SD of clinical parameters at baseline and three months after treatment using a paired t-test *p<.05: Significant level

Clinical parameters	Groups	Mean ± SD	p value
Plaque Index*	Group II	0.94±0.08	0.0031*
	Group III	0.60±0.20	
Gingival Index	Group II	0.67±0.21	0.1289
	Group III	0.94±0.34	
Bleeding on probing	Group II	1.02±0.31	0.2140
	Group III	1.79±0.29	
Pocket depth	Group II	1.88±0.54	0.7688
	Group III	1.99±0.71	
Clinical attachment level	Group II	3.06±1.05	0.6100
	Group III	2.73±1.12	

Glycemic status measured using fasting blood sugar (FBS) and glycated hemoglobin (HbA1c) levels showed statistical significance between baseline and after treatment (p < 0.001) among all the study groups (Table 3). On intergroup comparison, statistical significance was observed between Group 1 and 3 and Group 2 and 3 (p < 0.001) compared with Group 1 and Group 2 (Table 4).

**Table 3:** Table showing the intra-group comparison of mean ± SD of fasting blood sugar (FBS) and glycated hemoglobin (HbA1c) levels at baseline and three months after treatment using a paired t-test *p<.05: Significant level

Groups	Fasting Blood sugar (FBS) Mean ± SD	Glycated hemoglobin (HbA1c) Mean ± SD
Group 1		
Mean value at baseline	82.8 ± 7.5	5.7± 1.2
Mean value after 3 months	79.9± 7	5.5± 0.7
p-value	.002*	< .001*
Group 2		
Mean value at baseline	100 ± 9.7	6.1 ± 0.7
Mean value after 3 months	91.5 ± 6.9	5.7 ± 0.6
p-value	< .001*	< .001*
Group 3		
Mean value at baseline	174.1 ± 56	9.1± 2.1
Mean value after 3 months	132.1 ± 31.3	7.6 ± 1.4
p-value	< .001*	< .001*

**Table 4:** Table showing the inter-group comparison of mean ± SD of fasting blood sugar (FBS) and glycated hemoglobin (HbA1c) levels at baseline and three months after treatment using a paired t-test *p<.05: Significant level

Clinical parameters	Groups	Mean ± SD	p value
FBS	Group I	79.9 ± 7	0.0052
	Group II	91.5 ± 6.9	
HbA1c	Group I	5.5 ± 0.7	0.63
	Group II	5-7 ± 0.6	
FBS	Group I	79.9 ± 7	< .001*
	Group III	132.15	
HbA1c	Group I	5.5 ± 0.7	< .001*
	Group III	7.6 ±1.4	
FBS	Group II	79.9 ±10.8	0.0053*
	Group III	132.15	
HbA1c	Group II	5.7±0.6	< .001*
	Group III	7.6 ±1.4	

Analysis of total protein, albumin, globulin, albumin/globulin (A/G) ratio and microprotein levels revealed statistical significance in all the three groups (p < 0.001) (Table 5), and on intergroup comparison, a significant difference was observed for total protein levels between Groups 1 and 3 (p <.001) (Table 6).

**Table 5:** Table showing the intra-group comparison of the mean ± SD of inflammatory markers and proteinuria levels at baseline and three months after treatment using a paired t-test *p<.05: Significant level

Groups	Total Protein Mean ± SD	Albumin (gm/dl) Mean ± SD	Globulin (gm/dl)	Albumin/Globulin (A/G) ratio Mean ± SD	Microprotein Mean ± SD
Group 1					
Mean value at baseline	7.0 ± 0.4	4.0 ± 0.3	2.9 ± 0.3	1.4 ± 0.2	15.8 ± 7.3
Mean value after 3 months	6.6 ± 0.4	3.6 ± 0.5	2.5 ± 0.4	1.4 ± 0.2	14.3 ± 6.3
p-value	< .001*	< .001*	< .001*	.0039*	< .001*
Group 2					
Mean value at baseline	7.1 ± 0.4	4.2 ± 0.3	2.8 ± 0.5	1.5 ± 0.3	20.8 ±12.8
Mean value after 3 months	6.7 ± 0.3	3.8 ± 0.4	2.4 ± 0.3	1.6 ± 0.2	15.8 ± 6.7
p-value	< .001*	< .001*	< .001*	.0031*	< .001*
Group 3					
Mean value at baseline	7.1 ± 0.4	4.1 ± 0.4	2.9 ± 0.3	1.4 ± 0.2	26.7 ± 2.7
Mean value after 3 months	6.6 ± 0.4	3.8 ± 0.3	2.6 ± 0.3	1.4 ± 0.2	14.9 ± 5.7
p-value	< .001*	< .001*	< .001*	.015*	< .001*

**Table 6:** Table showing the inter-group comparison of mean ± SD of total protein and microprotein levels at baseline and three months after treatment using a paired t-test *p<.05: Significant level

Clinical parameters	Groups	Mean ± SD	p value
Total Protein	Group I	6.4 ± 0.5	0.754
	Group II	6.7 ± 0.3	
Microprotein	Group I	14.3 ± 6.3	0.633
	Group II	15.8 ± 6.6	
Total Protein	Group I	6.4 ± 0.5	.0017*
	Group III	6.8 ± 0.4	
Microprotein	Group I	14.3 ±6.3	0.358
	Group III	14.8 ± 5.7	
Total Protein	Group II	6.7 ± 0.3	0.532
	Group III	6.8 ± 0.4	
Microprotein	Group II	15.8 ± 6.6	.08
	Group III	14.8 ± 5.7	

## Discussion

The association between diabetes and periodontal disease has been explored over the years by many researchers [10,11]. The frequency of periodontitis is more prevalent in diabetic individuals than in those without diabetes, and similarly, diabetic individuals with periodontitis have a six-fold higher risk for worsening of glycemic control over time compared to diabetic patients without periodontitis [12]. It was postulated that the chronicity, susceptibility, and substantive nature of periodontal pathogens may induce local cellular-mediated pathways to release numerous pro-inflammatory mediators, including cytokines. The presence of these pro-inflammatory mediators in the systemic circulation and at the tissue level promotes tissue insulin resistance in patients with diabetes mellitus [12,13]. Studies have also reported that these mediators influence the progression of type 2 diabetes mellitus directly by taking part in the insulin resistance pathway mechanism. Based on these assumptions and postulations, it was hypothesized that effective control and appropriate management of periodontal infection by prophylaxis and nonsurgical treatment could reduce the pathogen load and existing periodontal infection with metabolic control of diabetes mellitus [14,15].

In the present study, significant differences were observed in the assessed clinical periodontal parameters except for clinical attachment level in Group 1 after three months of non-surgical periodontal treatment (NSPT). Patients with chronic periodontitis and uncontrolled diabetes mellitus presented with substantial clinical attachment loss compared to non-diabetic individuals. Stöhr J et al., in a cross-sectional observational study, also revealed that the frequency of clinical attachment loss in diabetic subjects was twice that of those presented without diabetes [16]. In a similar cross-sectional study, Matthews DC also observed that diabetes mellitus affected all periodontal parameters, irrespective of the disease phase [17]. The improvement following non-surgical periodontal therapy in all the periodontal parameters assessed agreed with the observations obtained by Rodrigues DC et al. [18].

A significant decrease in the levels of FBS and HbA1c following nonsurgical periodontal therapy was observed in all three groups. Intergroup comparison between FBS and HbA1c levels after therapy revealed a significant reduction between Group 1 and Group 3 and also between Group 2 and Group 3, suggesting a direct relation between these parameters and Group 3. This could be attributed to the fact that periodontal therapy reduces periodontal pathogen load, release of pro-inflammatory cytokines, and systemic inflammation, thereby reestablishing tissue insulin sensitivity and thus improving the glycemic mechanism [19]. Iwamoto et al., in a study on the antimicrobial effect of periodontal therapy on circulating TNF-α (Tumor Necrosis Factor-Alpha) and HbA1c levels, revealed diminished systemic inflammatory levels with improved tissue insulin sensitivity and a restored glycemic control mechanism [14]. These results also established a direct association between periodontal treatment and low-grade inflammation and circulating pro-inflammatory cytokines, which can subsequently prevent the progression of diabetic nephropathy. Janket et al. also proposed that improving the health of periodontal tissues can certainly promote mechanisms involved in pathways of glycemic control [20].

In this study comparing the mean values of total protein, albumin, globulin, and microprotein within the groups, there was a statistically significant reduction witnessed in all three groups after three months of non-surgical periodontal treatment follow-up. However, when intergroup comparison was carried out for total protein and microprotein, a statistically significant reduction was observed for total protein only between Group 1 and Group 3, indicating the association between uncontrolled diabetes and chronic periodontitis in the development of proteinuria. Catena C et al., in an observational study, have shown that an increase in levels of acute-phase reactants and pro-inflammatory mediators, predominantly cytokines, may directly alter juxta-glomerular filtration activity, resulting in albuminuria [21]. Previous studies have also shown an increase in plasma concentration of high-sensitive C-reactive Reactive Protein (HS-CRP) in patients with overt albuminuria than in those with nor-albuminuria.

Studies have established a two- to three-fold increase in the risk of macroalbuminuria, an end-stage renal disease, with an 8.5 times higher mortality rate in individuals presenting with diabetic nephropathy caused by uncontrolled diabetes associated with severe chronic periodontitis [21,22]. Similarly, in the present study, a linear correlation was identified between urinary albumin excretion levels and periodontal disease inflammatory levels, suggesting a significant role of periodontal pathogen-induced pro-inflammatory systemic markers in the progression of diabetic nephropathy [19,20]. Despite similar research that has been done earlier, this study slightly differs from the others in that it is performed in type 2 diabetic patients with chronic periodontitis to see the effect of nonsurgical periodontal therapy on the levels of total protein, albumin, globulin, microprotein, and glycemic status using FBS and HbA1c levels. The observations of this study revealed significant improvement in clinical parameters with a significant reduction in the FBS and Hba1C values after non-surgical periodontal therapy. When intragroup comparison was done, there was statistical significance observed for total protein, albumin, globulin, and microprotein, also suggesting the reduced burden of the complications associated with diabetes.

Limitations

Several systematic observations among diverse populations are needed to sustain the existing evidence that nonsurgical periodontal therapy can largely influence the glycemic control mechanism and possibly aid in the reduction of the complications associated with diabetes. Similar intervention studies are further needed to clinically demonstrate improved glycemic control supported by a histopathological reduction in levels of inflammatory cells following periodontal therapy to support this hypothesis.

## Conclusions

Diabetes mellitus is a metabolic disorder manifested by abnormally high levels of blood glucose. The pro-inflammatory cytokine-mediated response in periodontal disease can contribute to the overall low-grade inflammatory reaction that occurs in diabetes and is characterized by chronic activation of innate resistance, adversely affecting glycemic control. Periodontal therapy with non-surgical treatment may reestablish tissue insulin sensitivity and increase glycemic control mechanisms by decreasing periodontal pathogens and serum levels of pro-inflammatory markers, predominantly cytokines. The reduction of proteinuria after periodontal therapy also proves the efficiency of the treatment in reducing the overall complications of diabetes, either directly or indirectly. As evidence of the close association between periodontal disease and diabetes continues to hoard, general physicians, endocrinologists, and oral health professionals should create a platform to work in association, thus improving overall general health and oral health care and bringing about effective glycemic control to avoid complications among patients with diabetes. Further, longitudinal studies are recommended to support the results of the present study.
